# Supplementary material for: Content-rich biological network constructed by mining PubMed abstracts
Source: BMC Bioinformatics. 2004 Oct 8;5:147. doi: 10.1186/1471-2105-5-147 (PMC528731; doi:10.1186/1471-2105-5-147)
Supplement: Additional File 2 — The original results of the above study (non-essential files are deleted to keep the file size under the limit set by BMC bioinformatics). [file 1471-2105-5-147-S2.bz2 › chilibotAdditionalFile2/dip20/347ID7721786E4495/html/IL4_IL4R.html]

 


 **IL4** and **IL4R** 
  
Found 5329 abstracts in PubMed, retrieved 20.  
 

 What does Google say? 
 PDF only 
| .edu only 

---

**Interactive relationship** (e.g. stimulation, inhibition, etc)

**Neutral relationship**- Lectins interact differentially with purified human eosinophils, cultured cord blood derived mast cells and the myeloid leukaemic cell line AML14.3D10 induction of interleukin 4  [ **IL4R** ]   [ **IL4** ]  secretion is conserved among granulocytes, but is not proportional to agglutination or lectin glycoprotein interaction.  Ref: 12859449 Clin Exp Allergy, 2003

**Non-interactive relationship** (e.g. studied together, co-existance, homology, etc.)

- A molecule solves psoriasis ? Systemic therapies for psoriasis inducing interleukin 4  [ **IL4R** ]   [ **IL4** ]  and Th2 responses.  Ref: 12879154 J Mol MedJ Mol Med, 2003
- In vitro culture of bone marrow BM cells with granulocyte macrophage colony stimulating factor and interleukin 4  [ **IL4R** ]   [ **IL4** ]  induced a dramatic increase of telomerase activity accompanied with their differentiation into DCs.  Ref: 12885944 J Leukoc Biol, 2003
- Changes in Expression of Transferrin, Insulin like Growth Factor 1, and Interleukin 4  [ **IL4R** ]   [ **IL4** ]  Receptors after Irradiation of Cells of Primary Malignant Brain Tumor Cell Lines.  Ref: 12859234 Radiat Res, 2003
- In vitro stimulation of lymphocytes from both Th1 biased C57Bl 6 and T cell receptor transgenic mice and Th2 biased BALB c mice in the presence of OdDHL demonstrated that OdDHL inhibits in vitro cytokine production in response to both mitogen and antigen, with gamma interferon IFN gamma tending to be more inhibited than interleukin 4  [ **IL4R** ]   [ **IL4** ]  IL 4  [ **IL4** ] .  Ref: 12874321 Infect Immun, 2003
- Interleukin 4  [ **IL4R** ]   [ **IL4** ]  levels significantly decreased P <.  Ref: 12877453 Ann Allergy Asthma Immunol, 2003
- In view of the importance of interleukin 4  [ **IL4R** ]   [ **IL4** ]  IL 4  [ **IL4** ]  gene as a genetic factor for adult periodontitis, we investigated the relationship between two polymorphisms 590 C > T polymorphism and 70 bp repeat polymorphism of the human IL 4  [ **IL4** ]  gene and adult periodontitis in the Korean population.  Ref: 12877559 Arch Pharm Res, 2003
- Expression of transferrin receptors, type 1 insulin like growth factor receptors IGF1R, and interleukin 4  [ **IL4R** ]   [ **IL4** ]  receptors  **IL4R**  was measured by flow cytometry analysis on unirradiated cells and on cells 3 to 120 h after irradiation.  Ref: 12859234 Radiat Res, 2003
- Two polymorphisms of interleukin 4  [ **IL4R** ]   [ **IL4** ]  gene in Korean adult periodontitis.  Ref: 12877559 Arch Pharm Res, 2003
- APN CD13 was significantly upregulated by treatment with interleukin 4  [ **IL4R** ]   [ **IL4** ]  IL 4  [ **IL4** ] , interferon gamma IFNgamma, and the glucocorticoids dexamethasone and hydrocortisone.  Ref: 12861419 Arch Dermatol ResArch Dermatol Res, 2003
- With splenocytes from vaccinated mice, specific T cell responses, as well as gamma interferon and interleukin 4  [ **IL4R** ]   [ **IL4** ]  production, were observed after stimulation with P. chabaudi adami infected erythrocytes, demonstrating the specificity of genomic library vaccination for two of the three libraries constructed.  Ref: 12874330 Infect ImmunInfect Immun, 2003
- When examined in the presence of both CD154 and interleukin 4  [ **IL4R** ]   [ **IL4** ] , Tax had a clear supportive role on B cell growth, with an impact on B cell proliferation, cell cycle phase distribution, and survival.  Ref: 12881710 Oncogene, 2003
- Interleukin 4  [ **IL4R** ]   [ **IL4** ]  IL 4  [ **IL4** ]  is a major cytokine regulating IgE production and IgE response.  Ref: 12872985 Mol Cells, 2003
- METHODS Interferon gamma IFN gamma and interleukin 4  [ **IL4R** ]   [ **IL4** ]  IL 4  [ **IL4** ]  secreted by CD4 T cells were detected by flow cytometry in 40 patients with CA and 20 healthy volunteers, and significance of Th1 Th2 cell ratio in the etiology of the CA was analyzed.  Ref: 12865237 Di Yi Jun Yi Da Xue Xue Bao, 2003
- Real time polymerase chain reaction studies revealed significantly higher interleukin 4  [ **IL4R** ]   [ **IL4** ]  p< ; 0.05 and transforming growth factor beta p< ; 0.05 gene expression in the gastric mucosa in infected mice than in uninfected mice at both two and six months after AIG induction.  Ref: 12865266 Gut, 2003
- The roles of interleukin 4  [ **IL4R** ]   [ **IL4** ]  IL 4  [ **IL4** ]  and IL 13 in the regulation of immunity to Leishmania donovani infection are still poorly understood.  Ref: 12874364 Infect ImmunInfect Immun, 2003
- Interleukin 4  [ **IL4R** ]   [ **IL4** ]  IL 4  [ **IL4** ]  is crucial for the differentiation of naive T helper T H cells into the T H 2 effector cells that promote humoral antibody immunity and provide protection against intestinal helminths.  Ref: 12876556 Nat Rev Immunol, 2003
- In the ceramic on ceramic group, leukocytes and lymphocyte subsets were not significantly changed, but a significant increase in type 2 cytokines restored the ratio of interferon gamma to interleukin 4  [ **IL4R** ]   [ **IL4** ]  to normal values.  Ref: 12892206 J Bone Joint Surg Br, 2003
- Interleukin 4  [ **IL4R** ]   [ **IL4** ]  and Transforming Growth Factor beta Have Opposing Regulatory Effects on Gamma Interferon Mediated Inhibition of Cryptosporidium parvum Reproduction.  Ref: 12874337 Infect Immun, 2003
- Changes in Expression of Transferrin, Insulin like Growth Factor 1, and Interleukin 4  [ **IL4R** ]   [ **IL4** ]  Receptors after Irradiation of Cells of Primary Malignant Brain Tumor Cell Lines.  Ref: 12859234 Radiat Res, 2003
- Both Interleukin 4  [ **IL4R** ]   [ **IL4** ]  IL 4  [ **IL4** ]  and IL 4  [ **IL4** ]  Receptor alpha Signaling Contribute to the Development of Hepatic Granulomas with Optimal Antileishmanial Activity.  Ref: 12874364 Infect ImmunInfect Immun, 2003
- undetectable interleukin 4  [ **IL4R** ]   [ **IL4** ]  and interleukin 10.  Ref: 12891535 Gastroenterology, 2003
- Neutralizing antibodies against human RANTES, MIP 1alpha, MIP 1beta, alpha interferon IFN alpha, IFN beta, IFN gamma, interleukin 4  [ **IL4R** ]   [ **IL4** ]  IL 4  [ **IL4** ] , IL 10, IL 13, IL 16, MCP 1, MCP 3, tumor necrosis factor alpha TNF alpha, or TNF beta failed to reverse the HIV 1 suppressive activity.  Ref: 12885891 J VirolJ Virol, 2003
- Using an in vitro cell culture system, we investigated how the key regulatory cytokines interleukin 4  [ **IL4R** ]   [ **IL4** ]  IL 4  [ **IL4** ] , IL 10, IL 13, and TGF beta might modulate the effect of IFN gamma in inducing resistance to infection in enterocyte cell lines.  Ref: 12874337 Infect Immun, 2003
- Interleukin 4  [ **IL4R** ]   [ **IL4** ]  induces Coxiella burnetii replication in human monocytes but not in macrophages.  Ref: 12860673 Ann N Y Acad Sci, 2003
- Before THR, the patients showed a decrease in leukocytes and myeloid cells in comparison with healthy donors, and a prevalence of type 1 T lymphocytes, which was confirmed by the increase in ratio of interferon gamma to interleukin 4  [ **IL4R** ]   [ **IL4** ] .  Ref: 12892206 J Bone Joint Surg Br, 2003
